# Habitat‐Driven Sexual Dimorphism in *Triplophysa strauchii* Between Oxbow Lake and Stream: A Geometric Morphometric Analysis

**DOI:** 10.1002/ece3.73666

**Published:** 2026-05-11

**Authors:** Yan Li, Ya‐Han Meng, Wei‐Zhen Gao, Lei Shi

**Affiliations:** ^1^ Xinjiang Key Laboratory for Ecological Adaptation and Evolution of Extreme Environment Organism, College of Life Sciences Xinjiang Agricultural University Urumqi China; ^2^ College of Animal Sciences Xinjiang Agricultural University Urumqi China

**Keywords:** body shape analysis, microhabitat, natural selection, niche divergence, operculum, sexual selection

## Abstract

Sexual dimorphism is widespread among fish species, yet the ecological factors shaping this phenomenon remain poorly understood. Using traditional morphometric analyses of 319 specimens together with geometric morphometrics, this study examined sexual dimorphism in *Triplophysa strauchii* across two basins. The results showed that both the 42 specimens from the oxbow lake habitat in DL and the 34 specimens from the sedimentary‐bottom stream in LS exhibited male‐biased sexual size dimorphism (SSD). Males had larger body size, larger heads, and greater caudal peduncle depth, traits that may enhance locomotor and foraging performance. These findings suggest that sexual selection is an important mechanism underlying sexual dimorphism in *T. strauchii* across different river basins. The operculum also exhibited sexual differences across basins: females had narrower opercula, whereas males had more rounded and obtuse opercula. Canonical variate analysis (CVA) based on Procrustes distances further indicated that sexual dimorphism in opercular shape was more pronounced in DL than in LS. Differences in eye size were also observed: females from Dacao Lake had larger eyes than males, whereas males from Liutiao Stream had larger eyes than females. Together, these results reveal fine‐scale variation in fish sexual dimorphism in response to diverse selective forces (these inferences are based on habitat differences and previous studies, not direct field measurements).

## Graphical abstract

The combined effects of sexual selection and natural selection give rise to sexual dimorphism in operculum and eye morphology of *T. strauchii* across the two river basins. And our study reveal the fine‐scale responses of fish sexual dimorphism to diverse selective forces.

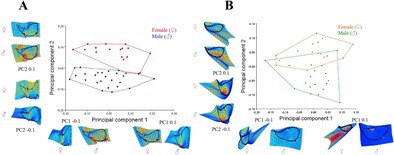

## 1 Introduction

Sexual dimorphism (SD) refers to differences between male and female individuals of the same species, including body size, specific morphological traits, coloration, physiology, and behavior. These differences may result from the combined effects of ecological and evolutionary processes (Sudasinghe 2025). In fish, sexual dimorphism is mainly manifested as differences in body coloration, fin length, and specific skin ornamentation during the breeding period, such as nuptial coloration. Sexual size dimorphism (SSD), defined as differences in body size between the sexes, is the most common form of sexual dimorphism (Wearmouth and Sims 2008). In many freshwater fish species, females are generally larger than males (Magurran and Macias Garcia 2000). However, male–male competition and paternal care can also favor the evolution of larger males (Horne et al. 2020).

The mechanisms underlying the formation of sexual dimorphism mainly include three core hypotheses: the sexual selection hypothesis, the fecundity selection hypothesis, and the niche divergence hypothesis. The sexual selection hypothesis is typically used to explain male‐biased sexual dimorphism, in which larger body size may confer advantages in male–male contests and territory defense (Hedrick and Temeles 1989). Additionally, males with larger heads may exhibit higher feeding efficiency, enabling them to acquire more net energy (Hu et al. 2017). This pattern is commonly observed in species exhibiting nest guarding or sperm competition (Kang et al. 2025). In addition, females may prefer males with larger body size or more prominent secondary sexual characteristics, because these traits may reflect male genetic quality or resource acquisition ability. The fecundity selection hypothesis is generally employed to explain female‐biased sexual dimorphism, positing that females evolve increased abdominal capacity to support greater egg production. This allows females to produce more or larger eggs or offspring, thereby maximizing offspring fitness (Fitch 1970) and increasing overall reproductive output to ensure species persistence (Hu et al. 2017). Additionally, larger female body size enables individuals to exploit a wider range of food resources and accumulate greater energy reserves to meet the high metabolic demands associated with spawning and offspring care. Moreover, increased body size reduces predation risk, thereby facilitating successful reproduction. The niche divergence hypothesis does not strictly predict which sex should be larger, but proposes that, under natural selection, differences in nutritional niches drive trait differentiation between males and females. The primary drivers of this hypothesis are the scarcity of nutritional resources and increased intraspecific competition. Under this selective pressure, individuals with phenotypic traits that allow them to exploit different food resources or habitats gain a greater adaptive advantage. Specifically, differences in morphology or body size allow males and females to occupy different niches, thereby facilitating divergence in feeding behavior and habitat selection and promoting ecological differentiation. This helps alleviate intraspecific or intersexual competition, ultimately leading to sexual dimorphism in resource utilization (Walsh and Reznick 2008). Furthermore, factors such as genetic mechanisms, sex hormones, temperature, food abundance, population density, allometric growth, and the timing of tissue differentiation may all contribute to the expression of sexual dimorphism. Investigating SD in *T. strauchii* in relation to these factors can provide insights into the species' reproductive strategies, foraging behavior, and ecological adaptation, thereby informing conservation efforts that account for sex‐specific ecological pressures and habitat preferences (Kiliçli et al. 2025).

Geometric morphometrics has been increasingly applied to the study of phenotypic variation in animals. It is a method based on the analysis of geometric data, such as Cartesian coordinates, specific reference points, curves and contours to examine variations in the shape and size of anatomical structures (Kiliçli et al. 2025). Among the suite of analytical tools in this framework, landmark‐ and semi‐landmark‐based approaches represent the most widely utilized geometric morphometric methods (Mitteroecker and Schaefer 2022). Landmarks are discrete, homologous morphological points that can be precisely localized on each specimen, whereas semi‐landmarks are points placed at predefined intervals along curves between two fixed landmarks (Benítez 2013). This methodological framework uses landmark coordinates to quantify organismal shape; after eliminating the confounding effects of translation, scaling, and rotation, the retained shape‐specific geometric information can be further visualized and analyzed using thin‐plate spline grids and relative warp analyses to characterize shape differences among samples (Hernandez et al. 2022). By applying geometric morphometrics to quantify and visualize sexual variation in curved surface morphology, the interference of body size in *T. strauchii* is minimized, allowing for more objective and precise detection of sexually dimorphic traits. It thus reveals subtle and complex morphological differences between the sexes, effectively identifying sex‐specific traits that are often undetectable using traditional linear measurements (Hernandez et al. 2022; Günay et al. 2025). Therefore, it provides insights into the evolutionary and developmental mechanisms underlying phenotypes and strengthens the integration of morphological and functional analyses (Zelditch et al. 2012; Liu et al. 2025). In contrast, traditional morphometrics quantifies external morphology by measuring and statistically analyzing linear traits and their derived ratios (Liebl et al. 2021; Marcos et al. 2016).

The genus *Triplophysa* (Cypriniformes: Nemacheilidae) represents a species‐rich and taxonomically intricate clade within the family Nemacheilidae (Li et al. 2017). Adapted to diverse habitats, *T. strauchii* thrives in cold alpine environments and often serves as a dominant and ecologically important taxon within its distribution range (Zhang et al. 2024). *T. strauchii* is primarily distributed in Xinjiang, China, where it inhabits numerous rivers on the northern slopes of the Tianshan Mountains (Guo et al. 2008). To date, research on *T. strauchii* remains relatively limited, with existing studies focusing mainly on its biological characteristics (Guo et al. 2002, 2008) and mitochondrial genome characterization (Han et al. 2016). Moreover, its wide distribution across distinct water bodies provides a natural comparative framework for evaluating how environmental pressures shape morphological differences between the sexes. Therefore, *T. strauchii* represents an ideal non‐model species for testing these three hypotheses.

As a minerotrophic peatland dominated by aquatic plants, Dacao Lake exhibits strong water‐retention and purification capacities through pollutant filtration and adsorption. In addition, the Baiyang River maintains sufficient dissolved oxygen levels, ranging from 5 to 6 mg/L, and its gentle flow velocity facilitates the sedimentation of suspended solids, thereby resulting in clear water quality (Pang et al. 2005). In contrast, Liutiao Stream is a sediment‐bottomed stream with a substrate dominated by gravel and sand, interspersed with localized deposits of clay or fine silt.

Across different habitat types and resource availability regimes, *T. strauchii* exhibits distinct growth traits. In natural habitats characterized by abundant food resources and the absence of competition from invasive species, this species maintains a complete age‐class structure and displays a rapid growth pattern, representing an adaptive phenotype reflecting its ecological specialization to high‐elevation environments. In contrast, in stream habitats subjected to biological invasion pressure and interspecific competition, its growth rate is reduced, accompanied by smaller body size and a demographic shift toward younger age classes (Meng et al. 2025).

Applying geometric morphometrics to analyze sexual dimorphism in fish aids in understanding sex determination mechanisms and provides key insights into species identification, conservation, and evolutionary biology. Given the significant differences in substrate and flow velocity between the two basins, we predict that:

1. In slow‐flowing oxbow lake environments, morphological differentiation between the sexes is predicted to be driven predominantly by sexual selection, such as male–male competition. Males are expected to evolve more exaggerated morphological traits (e.g., enlarged heads, elongated fins, or expanded tails), leading to stronger and more pronounced sexual dimorphism in overall body shape and specialized structural traits.
2. In fast‐flowing stream environments, morphological differentiation between the sexes is predicted to be driven predominantly by natural selection, which is expected to act mainly on swimming stability, hydrodynamic efficiency, and respiratory efficiency. Phenotypes adapted to lotic conditions are expected to constrain the evolution of sex‐specific morphological divergence, thereby resulting in weaker sexual dimorphism in body size and most external morphological traits.

## 2 Materials and Methods

### 2.1 Materials

**FIGURE 1:**
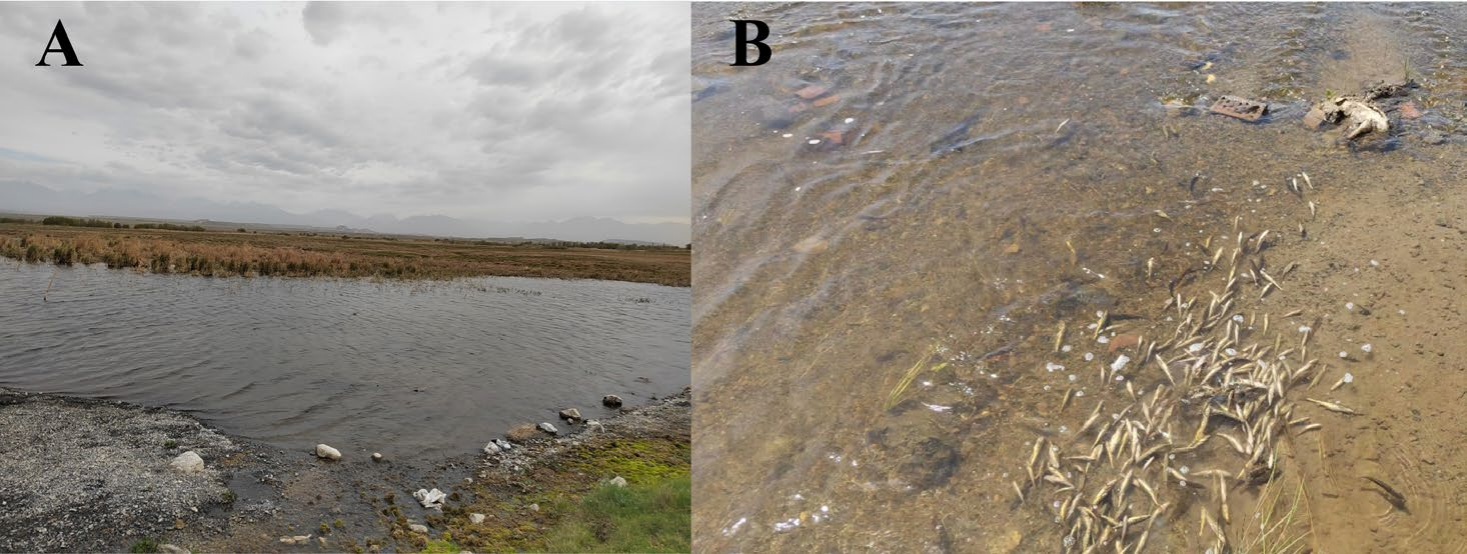
Habitats of Dacao Lake (A) and Liutiao Stream (B).

Dacao Lake (DL; 88°24′ E, 43°21′ N) is located in Daban Town, Urumqi, Xinjiang, and is an oxbow lake formed in the middle reaches of the Baiyang River (Figure 1A). DL is characterized by a small water surface area, a short connecting channel to the main river course, and an elevation of 1169 m (Zhang et al. 2021). Liutiao Stream (LS; 92°59′ E, 43°39′ N) is situated in Barkol County, Hami City, Xinjiang, and serves as a major tributary within the Barkol Lake basin (Figure 1B). And it lies in the Barkol Valley, which is bounded by the northern slope of the Barkol Mountains and the southern slope of the Moqin Ula Mountains. LS is characterized by a low flow velocity, short channel length, and substantial seepage losses, with an elevation of 1650 m.

*T. strauchii* used in this study were collected in May 2024 from Dacao Lake (DL; *n* = 218) and Liutiao Stream (LS; *n* = 101) using cage nets (dimensions: 5 m length × 3 m width; mesh size: 4 mm). Reproductive maturity was confirmed by gonadal examination, in which mature females exhibited enlarged, yellowish to orange, opaque ovaries and mature males displayed elongated, whitish to creamy testes with smooth or lobed texture (Agger et al. 1974; Núñez and Duponchelle 2009). We selected size‐matched and well‐preserved adult specimens that had similar body lengths to minimize allometric effects and showed no deformation or missing anatomical structures for geometric morphometric analysis (Bookstein 1991; Cadrin 2000): 17 females and 25 males from the Dacao Lake (DL) (23 of which were for fish body research), and 19 females and 15 males from the Liutiao Stream (LS). All experiments and animal handling were conducted according to research protocols approved by the Animal Welfare and Ethics Committee of Xinjiang Agricultural University.

### 2.2 Traditional Morphometric Analysis

Specimens of *T. strauchii* preserved in 10% formaldehyde solution were placed in a tray and measured using a mechanical vernier caliper (accuracy: 0.01 mm) and an electronic balance. A total of 18 measurable morphological traits was as follows (Wu and Wu 1992): total length (TL), body length (BL), head length (HL), head height (HH), long torso (LT), tail length (TAL), snout length (SNL), eye diameter (ED), postorbital head length (PHL), body depth (BD), caudal‐peduncle depth (CPD), dorsal fin length (DL), caudal fin length (CFL), pelvic fin length (VL), anal fin length (AL), pectoral fin length (PL), Eye interval (EI), body width (BWI), and body weight (BW). All of the measurements were taken by a single observer and that random individuals were measured repeatedly.

### 2.3 Image Acquisition and Processing for Geometric Morphometric Analysis

Specimens with intact morphological preservation were selected for digital imaging. Each specimen was secured on an M5 hex socket screw against a black background (surface was smooth, with only gentle pressure applied and positioned away from landmarks to avoid tissue damage and morphological distortion) or pinned onto a white polystyrene base to ensure natural body extension and full expansion of all fins. Using a Canon EOS 90D camera with an EF 60 mm lens mounted on a tripod at a fixed height, each specimen was photographed vertically from head to tail at a standardized position. A scale ruler was included in each photograph for calibration (Zelditch et al. 2012), and all imaging procedures were performed by the same operator to ensure consistency.

### 2.4 Geometric Morphometric Analysis

#### 2.4.1 Establishment and Selection of Landmarks and Semi‐Landmarks

**FIGURE 2:**
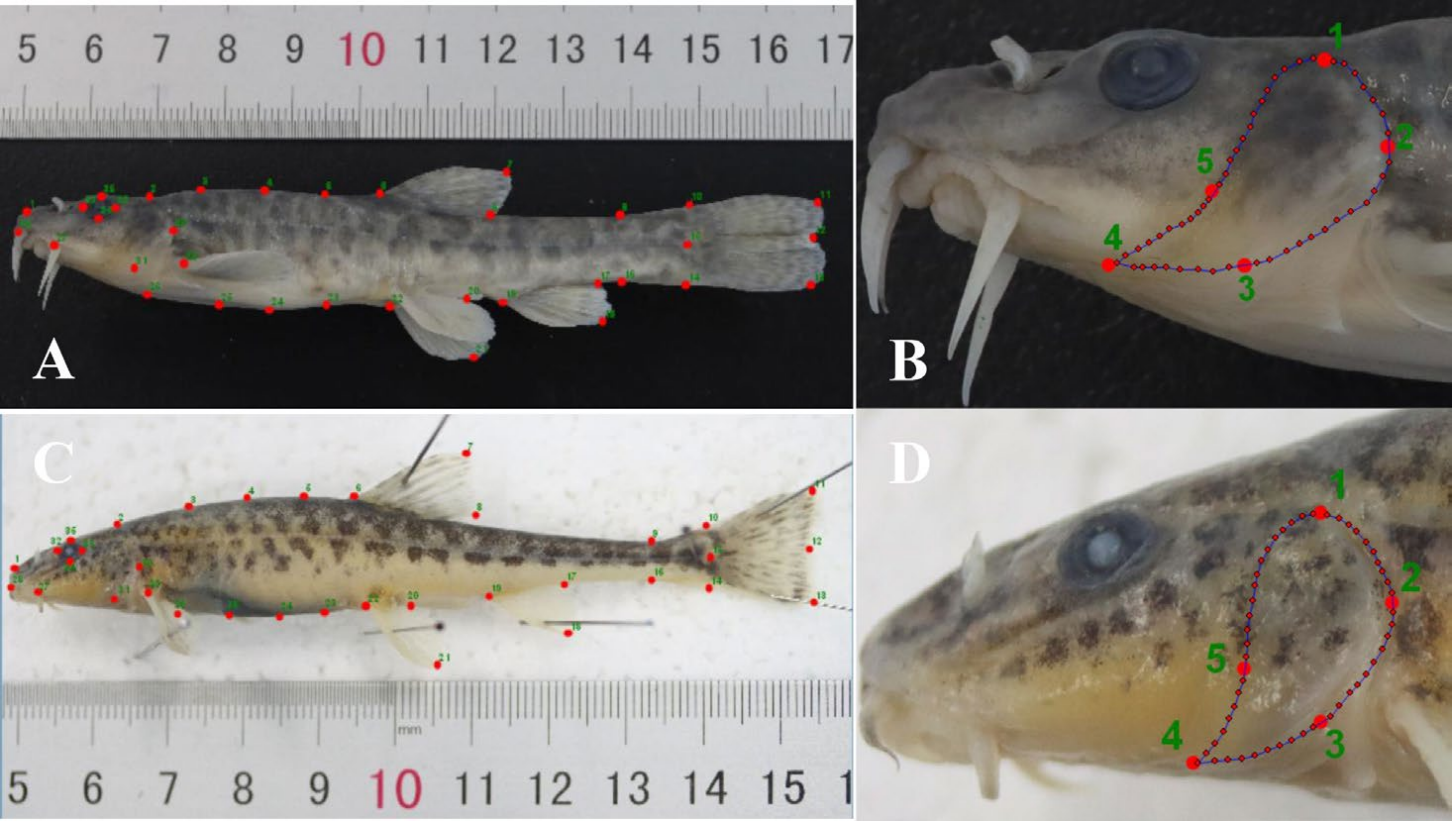
Establishment and selection of landmarks and semi‐landmarks from Dacao Lake (A, fish body, B, operculum) and Liutiao Stream (C, fish body, D, operculum).

Images were digitized using TPSDig232 software (Rohlf 2015), with the resulting coordinate data exported in TPS file format. For whole‐body morphological analysis, 35 landmarks were digitized for each sex of *T. strauchii* , including 15 landmark I, 10 landmark II, and 10 landmark III (Table 1; Figure 2A,C). For operculum analysis, 5 landmarks and 51 Semi‐landmarks were selected for each sex (Figure 2B,D). Landmarks I are defined as intersections between different tissues, such as the contact points between fish fins and the body. Landmarks II refer to distinct depressions or projections on anatomical structures, including the caudal fork notch, bony processes, and other clearly identifiable protrusions. Landmarks III represent extremal points of structures, defined relative to other landmarks, such as the widest points (Bookstein 1991; Kiliçli et al. 2025). Furthermore, five specimens were randomly selected from each group for repeated landmark digitization, resulting in a total of 20 samples for both sexes across the two basins.

**TABLE 1:** Types and definitions of landmarks on the fish body.

Landmark types	Definition	Landmark types	Definition
Landmark I			
1	Tip of the snout	5	Dorsal perpendicular point at body length midpoint
6	Anterior end of the dorsal fin base	8	Posterior end of the dorsal fin base
10	Dorsal end of the caudal fin base	14	Ventral end of the caudal fin base
17	Posterior end of the anal fin base	19	Anterior end of the anal fin base
20	Posterior end of the pelvic fin base	22	Anterior end of the pelvic fin base
23	Ventral perpendicular point at the midpoint of body length	24	Ventral perpendicular point at the highest prominence of the mid‐trunk region
25	Ventral perpendicular point at the maximum curvature of the anterior trunk region	26	Junction point of the operculum and mandible
29	Origin of the pectoral fin		
Landmark II			
2	Posterior end of the occipital bone	15	Posterior end of the caudal peduncle
27	Base of the outer rostral barbel	28	Base of the inner rostral barbel
30	Posterior margin of the operculum	31	Ventral origin of the preoperculum
32	Anterior margin of the orbit	33	Posterior margin of the orbit
34	Ventral margin of the orbit	35	Dorsal margin of the orbit
Landmark III			
3	Maximum curvature of the anterior trunk region	4	Highest prominence of the mid‐trunk region
7	Highest point of the dorsal fin	9	Narrowest point of the dorsal caudal peduncle
11	Dorsal‐most point of the caudal fin	12	Fork point of the caudal fin
13	Ventral‐most point of the caudal fin	16	Narrowest point of the ventral caudal peduncle
18	Lowest point of the anal fin	21	Lowest point of the pelvic fin

#### 2.4.2 Landmark Vector Superposition, Wireframe Construction and Deformation Visualization of Fish Body

Methods for visualizing fish body deformation include vector displacement analysis and thin‐plate spline (TPS). TPS‐formatted data from four fish groups were imported into the gmShiny v0.1.4 website (gmshiny.chatham.edu) (Baken et al. 2021). A generalized Procrustes analysis (GPA) was applied to the Cartesian coordinates of landmarks to perform translation, rotation, and scaling, fitting them to a uniform centroid size. This superimposition removes non‐shape‐related variation (Hernandez et al. 2022; Hammer et al. 2001), resulting in a vector superimposition plot of the 35 landmarks.

Based on the standardized data, vector analysis is conducted. The deformation positions of both sexes of the black‐spotted highland loach in different river basins are obtained according to the direction and magnitude of the vectors. The four TPS files were then analyzed in PAST3 (Version 1.0.0.0) using thin‐plate spline (TPS) to visualize deformation grids along principal component axes, which visualize the magnitude, direction, and distribution of overall and local shape differences by warping a regular grid from a reference form to a target form using thin‐plate spline interpolation (Bookstein 1991). Data for both sexes from Dacao Lake and Liutiao Stream were merged into two Tps files. And with a grouping table established. Principal component analysis (PCA) was conducted on the website https://www.chiplot.online/ to generate scatter plots of the first two principal components. Finally, MorphoJ 1.08.02 (Klingenberg 2011) was used to create wireframe diagrams that visualize differences between groups based on PCA results, which are structural frameworks constructed by connecting homologous landmarks with straight lines to clearly show relative displacements between landmarks and local morphological changes (Alsaigh and Alrashdi 2023; Zelditch et al. 2012).

#### 2.4.3 Thin‐Plate Spline and Principal Component Analyses of Operculum

For the four groups of operculum coordinate data, semi‐landmark data were first converted to landmark format using TpsUtil32 software (Rohlf 2015). Subsequently, opercular coordinate data of both sexes from the two river basins were merged into two composite TPS files. These merged datasets were then imported into MorphoJ (Version 1.08.02) to perform Procrustes Analysis for the average shape, and PCA was conducted on the covariance matrix (Jolliffe 2011; Gordon et al. 2022), generating scatter plots of the first and second principal components for the operculum of both sexes. Finally, TPS data from females and males of *T. strauchii* from each river basin were imported into PAST3 (Version 1.0.0.0) software separately for thin‐plate spline (TPS) analysis, yielding grid deformation diagrams that visualize shape variation along the PC1 and PC2 axes for each of the four sample groups (sex‐specific data from two basins).

### 2.5 Statistical Analysis

First, for the traditional morphological data, 18 measurable morphological traits and 1 body weight parameter were recorded using Microsoft Excel 2019, with subsequent statistical analyses conducted in R (Version 4.4.0; R Core Team 2016). Shapiro–Wilk tests for normality tests and Bartlett's tests for homogeneity of variance were conducted (Zammuto 1984), and all data satisfied the requirements of normality and homoscedasticity. Differences in mean body length between sexes were evaluated with an Independent‐Samples *t*‐test. For comparisons of other morphological traits, a One‐Way Analysis of Covariance (ANCOVA) was performed, with body length included as a covariate to account for the influence of overall body size (Mishra et al. 2019). And to account for confounding effects of growth stage and body size, standard length was used as a covariate in Iinear regression, followed by Independent‐Samples *t*‐tests. Euclidean distances between sexes were then calculated to quantify morphological differences in eye morphology between the two basins (Hetherington et al. 2015).

To assess allometric growth between sexes, Reduced Major Axis Regression (RMA) was performed using the “sma” function in the “smatr” package (McArdle 1988; Abouheif and Fairbairn 1997), with each local morphological trait as the dependent variable and head‐body length as the independent variable (Warton et al. 2012). A slope heterogeneity test was conducted; a significant difference in slopes between sexes was interpreted as evidence of allometric growth. Descriptive statistics are reported as mean ± standard error, and the significance level was set at *α* = 0.05.

Principal component analysis (PCA) and Procrustes multivariate analysis of variance (PMANOVA) (Goodall 1991; Klingenberg et al. 2002) were employed to statistically evaluate the coordinate data of body and operculum shape between sexes of *T. strauchii* across different river basins. In MorphoJ (Version 1.08.02), standardized operculum landmark data were subjected to PMANOVA to determine the significance of morphological differences in operculum shape between sexes within the two basins. Also, the two sets of landmark coordinates from the 20 randomly selected specimens were imported into MorphoJ (Version 1.08.02) for Procrustes ANOVA to quantify digitization error.

Canonical variate analysis (CVA) was performed in MorphoJ (Version 1.08.02) to quantify the degree of sexual dimorphism in the operculum among different river basins. Results were characterized by Mahalanobis distances and Procrustes distances, with *p*‐values derived from 10,000 permutation replicates to quantify the statistical significance of morphological divergence between sexes. Procrustes distance, calculated as the distance between the mean female and male opercular shapes in morphospace, was used as a standardized metric to quantify shape dissimilarity (Dryden and Mardia 2016). The detailed procedure of the statistical analysis is shown in Table S14.

## 3 Results

### 3.1 Traditional Morphometric Analysis

#### 3.1.1 Sexual Dimorphism

Independent‐Samples *t*‐tests (Tables S1 and S2) showed that adult males of *T. strauchii* from both basins had significantly greater body length, body weight, and total length than females (*p* < 0.05). ANCOVA with body length as the covariate indicated that in the Dacao Lake population, males exhibited significantly larger caudal‐peduncle depth (*p* = 0.00319), body width (*p* = 0.027), eye interval (*p* = 0.0384), and head height (*p* = 0.0306) (*p* < 0.05) (male>female), whereas females had significantly larger eye diameter (*p* = 0.000782) (*p* < 0.001) (female>male). In the Liutiao Stream population, males displayed significantly larger eye diameter (*p* = 0.0251), dorsal fin length (*p* = 0.00607), pelvic fin length (*p* = 0.029), anal fin length (*p* = 0.012), pectoral fin length (*p* = 0.000), and head height (*p* = 0.0041) (*p* < 0.05) (male>female) compared to females. And sexual dimorphism in eye morphology was significant in both size‐related and size‐corrected values in DL (*p* = 0.003) (*p* < 0.05), and significant in size‐related values (*p* = 0.000) (*p* < 0.001) but marginally significant in size‐corrected residuals (*p* = 0.05) in LS (Table S12).

#### 3.1.2 Allometric Growth

**FIGURE 3:**
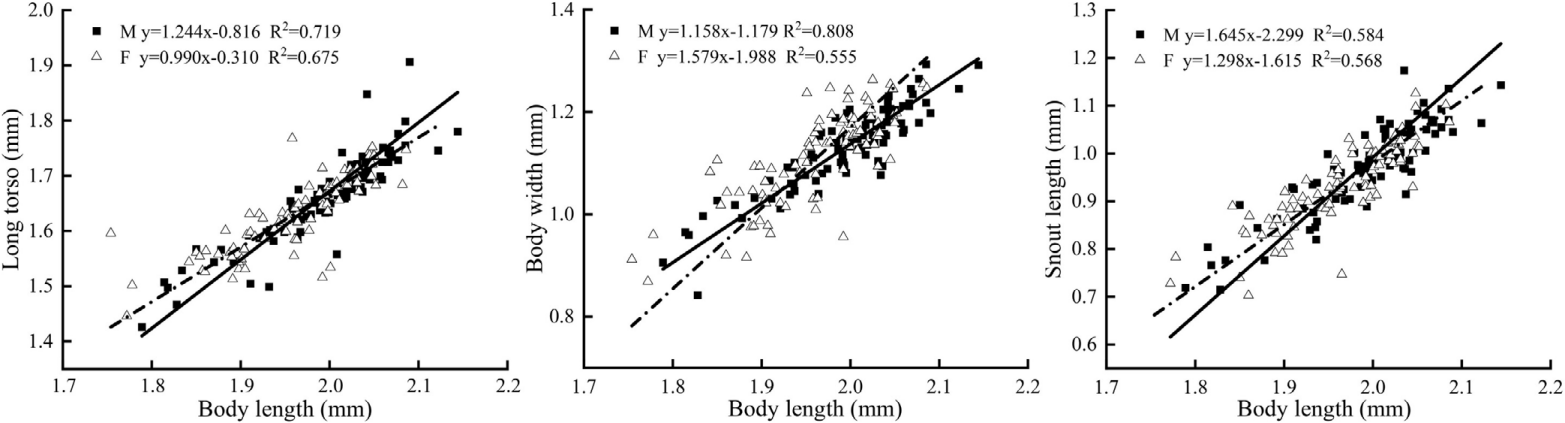
Regression of morphological characteristics and body length of *Triplophysa strauchii* in Dacao Lake.

**FIGURE 4:**
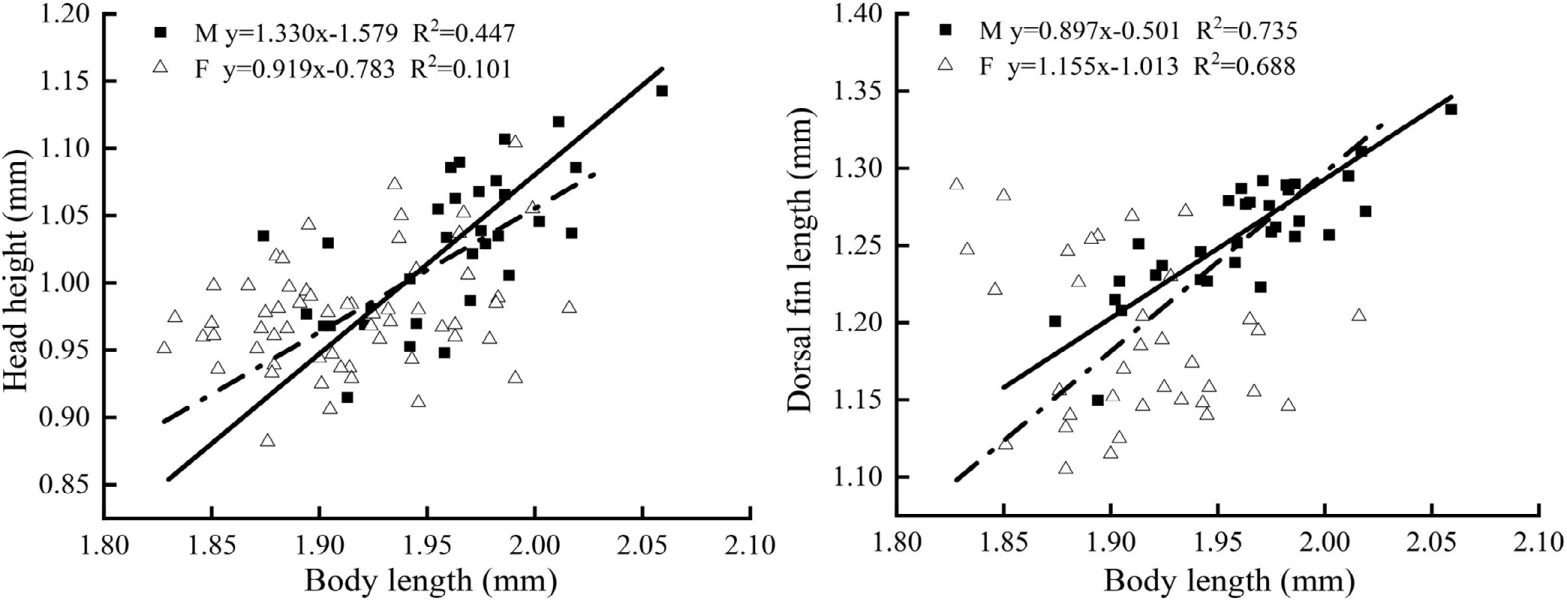
Regression of morphological characteristics and body length of *Triplophysa strauchii* in Liutiao Stream.

Reduced Major Axis Regression (RMA) showed that male *T. strauchii* from Dacao Lake grew significantly faster than females in long torso (*p* < 0.01) and snout length (*p* < 0.05), whereas females grew significantly faster than males in body width (*p* < 0.01) (Figure 3). In the Liutiao Stream population, head height increased significantly faster in males (*p* < 0.05), while dorsal fin length grew significantly faster in females (*p* < 0.05) (Figure 4).

### 3.2 Geometric Morphometric Analysis of Fish Body

#### 3.2.1 Sexual Dimorphism of Fish Body

**FIGURE 5:**
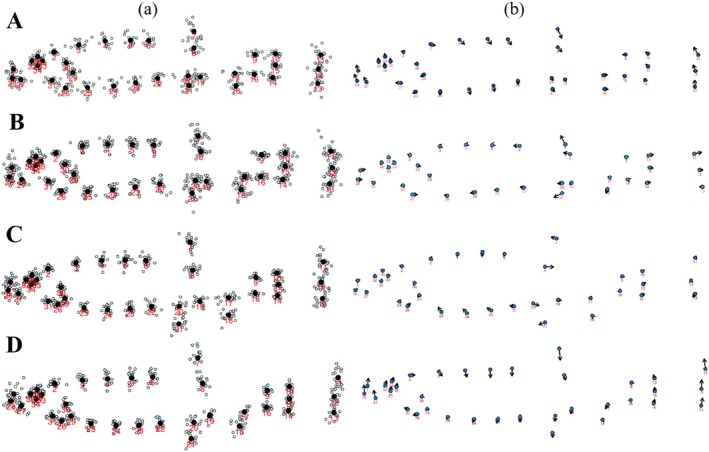
Landmark vector superposition plot (a) and relative warp analysis (b) of female (A‐Dacao Lake, C‐Liutiao Stream) and male (B‐Dacao Lake, D‐Liutiao Stream).

The superimposed landmark configurations of female and male *T. strauchii* are shown in Figure 5a. Principal component analysis of relative warps based on the mean shape revealed shape changes along discriminant axes, visualized using deformation vectors (Figure 5b). Vector direction and magnitude indicate that sexual dimorphism in the Dacao Lake (Figure 5A,B) was concentrated in the head (1, 26, 27, 28, 31), eye (32, 33, 34, 35), body (3, 4, 5, 23, 24), fins (6, 7, 8, 20, 21, 22), and caudal (10, 11, 12, 13). In the Liutiao Stream (Figure 5C,D), significant sexual shape variation occurred in the head (1, 2, 26, 27, 28), eye (32, 33, 34, 35), body (3, 4, 5, 23, 24, 25), fins (6, 7, 8, 19, 20, 21, 22), and caudal (11, 12, 13). Thus, sexual dimorphism in both basins is primarily expressed in the head (including eye and operculum), body, and caudal regions.

The wireframe diagram (Figure S1) indicates that the primary sexual dimorphism in *T. strauchii* across both river basins is concentrated in the head (eye and operculum) and the caudal regions. In the Dacao Lake, females (Figure S1A) exhibits a steeper anterior head profile and a more pronounced dorsal elevation at the shoulder, resulting in a more abrupt contour transition. The males (Figure S1B) show a gentler anterior head curvature with a smoother, more gradual contour without significant undulations. In the Liutiao Stream, females (Figure S1C) exhibit a slightly lower head position along the horizontal axis, yet demonstrate smooth transitional contours and a moderately elevated shoulder region. In contrast, males (Figure S1D) have a relatively higher head position, though the difference is subtle, warranting localized grid deformation analysis to characterize detailed shape variation. Regarding caudal morphology, the Liutiao Stream population possesses a narrower caudal peduncle and a more acute tail fin spread angle (the lines connecting landmarks 10–11–12), whereas the Dacao Lake population of both sexes shows a tail fin angle near 90°.

#### 3.2.2 Visualization of Sexual Dimorphism in Fish Body Across Different Basins

**FIGURE 6:**
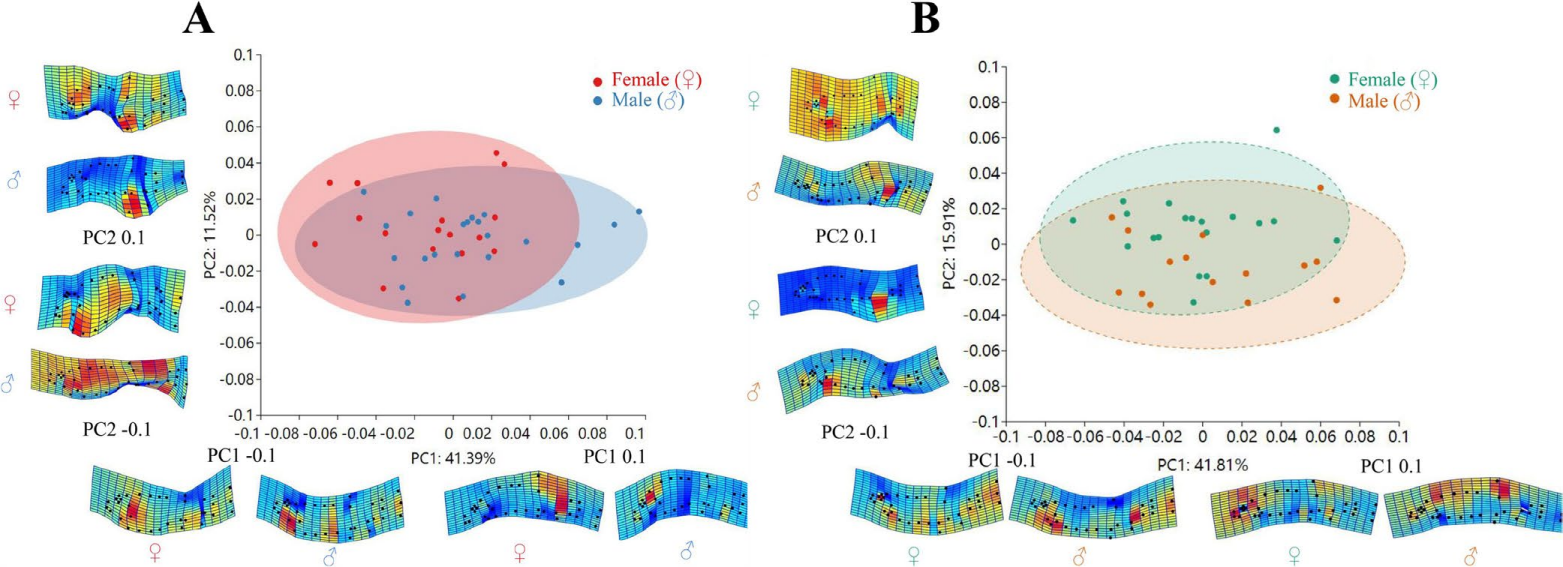
PCA scatter plot (PC1 vs. PC2) of body morphology for *Triplophysa strauchii* (male and female) from Dacao Lake (A) and Liutiao Stream (B).

The relative warp analysis for *T. strauchii* from Dacao Lake yielded 39 principal components, with PC1 (41.39%) and PC2 (11.52%) together explaining 52.91% of morphological variation between sexes (Figure 6A). For the Liutiao Stream population, 33 principal components were extracted; PC1 (41.81%) and PC2 (15.91%) collectively accounted for 57.72% of the total variance (Figure 6B). Scatter plots of PC1 and PC2 revealed substantial overlap yet distinct sexual clustering between sexes in both populations (Figure S4).

### 3.3 Geometric Morphometric Analysis of the Operculum

#### 3.3.1 Sexual Dimorphism of the Operculum

**FIGURE 7:**
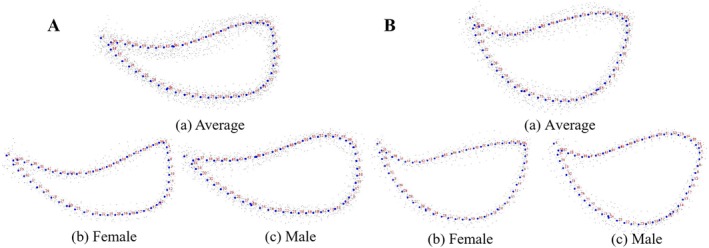
The average operculum morphology of *Triplophysa strauchii* (male and female) from Dacao Lake (A‐(a) Average (b) Female (c) Male) and Liutiao Stream (B‐(a)) Average (B‐(a) Average (b) Female (c) Male).

A least‐squares regression analysis was performed on opercular shape data of *T. strauchii* from both river basins using tpsSmall software. The results showed that the regression coefficients (slopes) between tangent space distances and Procrustes distances were 0.997 for females and 0.9957 for males in Dacao Lake, and 0.998 for females and 0.996 for males in Liutiao Stream. All four values approached 1, which verified the rationality and reliability of the selected landmarks and semi‐landmarks. In average operculum shapes, the male shape was closer to the overall average in Dacao Lake (Figure 7A(a)), and the Liutiao Stream (Figure 7B(a)) is generally broader and shorter than Dacao Lake. In both populations, females exhibit narrow operculum with sharper edges (Figure 7A,B(b)), while males (Figure 7A,B(c)) have rounder, blunter operculum. Grid deformation and vector analysis (Figure S5) revealed distinct sexual differences in landmark displacement patterns. In females (Figure S5A,C), landmarks 4, 36–43, and 45–53 shifted anteriorly with outward‐pointing vectors, while landmarks 1, 6–13, and 27–34 also moved anteriorly but with inward‐pointing vectors. In males (Figure S5B,D), the displacement patterns were opposite for the same landmark sets. The magnitude and direction of these vectors resulted in a more protruding anterior margin in females and a more pronounced posterior curvature in males.

These findings confirm that females of *T. strauchii* from both river basins possess a narrow operculum, whereas males have a rounder, blunter operculum.

#### 3.3.2 Visualization of Sexual Dimorphism in Operculum Across Different Basins

**FIGURE 8:**
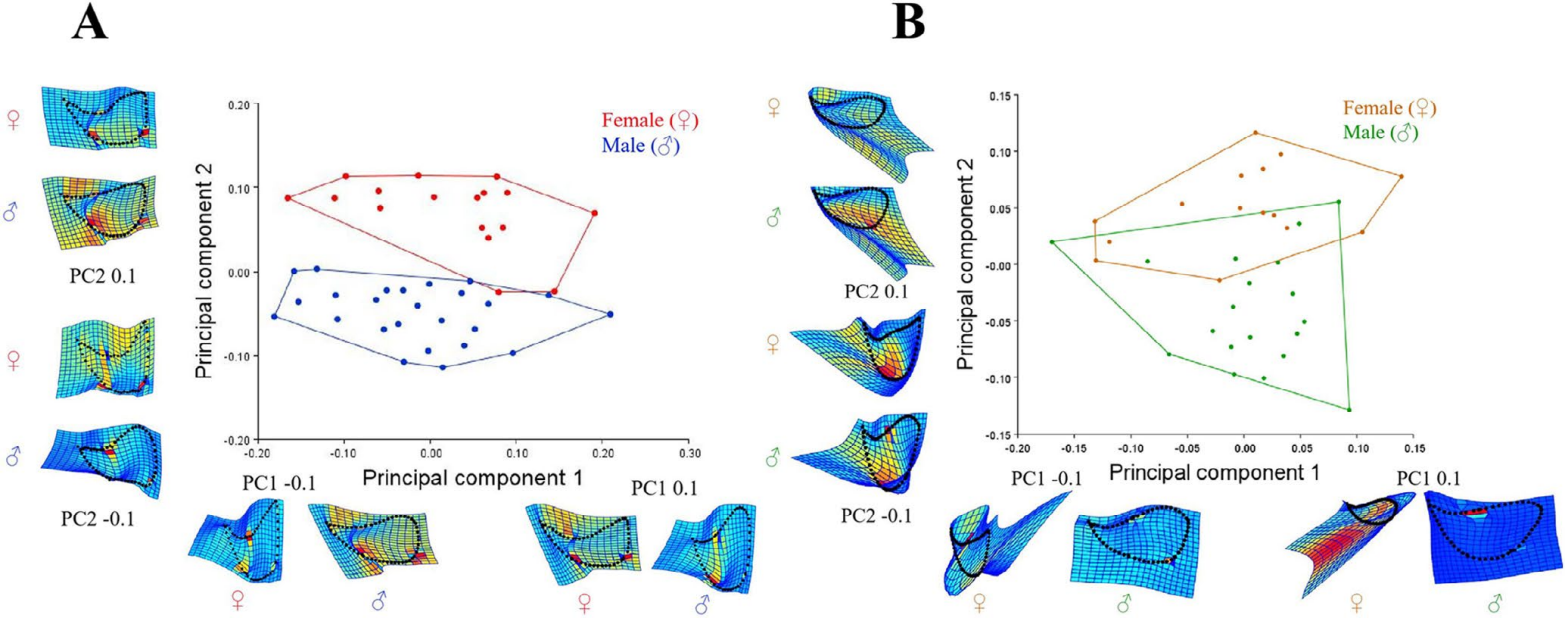
PCA scatter plot (PC1 vs. PC2) of operculum morphology for *Triplophysa strauchii* (male and female) from Dacao Lake (A) and Liutiao Stream (B).

Relative warp analysis of *T. strauchii* operculum extracted 41 principal components in Dacao Lake and 33 in Liutiao Stream, with cumulative variances of 69.96% (PC1 = 45.64%, PC2 = 24.33%) and 54.82% (PC1 = 29.78%, PC2 = 25.04%) respectively for components exceeding 10% contribution. Thus, PC1 and PC2 capture major sexual dimorphism in opercular shape in both populations. Scatter plots of PC1 and PC2 are presented for Dacao Lake (Figure 8A) and Liutiao Stream (Figure 8B). In both populations, the points show limited overlap between sexes and form two relatively distinct clusters, indicating sexual dimorphism in the operculum of *T. strauchii* . Scatter diagrams for four groups are provided in Figure S5.

#### 3.3.3 Procrustes Multivariate Analysis of Variance (PMANOVA)

Procrustes ANOVA results for Dacao Lake and Liutiao Stream showed no significant differences in operculum centroid size between sexes or among individuals (Tables S6 and S8) (*p* > 0.05). In contrast, highly significant sexual dimorphism was detected in operculum shape (Tables S7 and S9) (*p* < 0.001), indicating highly pronounced sexual shape dimorphism in the operculum of both basins. Meanwhile, digitization error accounted for only 0.62% of the total shape variation, which was substantially smaller than the biologically meaningful among‐individual variation (Table S11) (*F* = 16.03, *p* < 0.0001). For centroid size as well, digitization error accounted for only 2.86% of the total variation (Table S10) (*F* = 33.96, *p* < 0.0001).

#### 3.3.4 Degree of Sexual Dimorphism in Operculum of *T. strauchii* Across Different Basins

Canonical Variate Analysis (CVA) revealed that the Mahalanobis distances between sexes in operculum shape were 5.2714 for Dacao Lake and 4.4809 for Liutiao Stream, neither of which was significant based on 10,000 permutations (*p* > 0.05). And the Procrustes distances were 0.1273 and 0.0958 for the two populations, both of which exhibited highly significant differences based on 10,000 permutations (*p* < 0.001). Given the higher statistical sensitivity of Procrustes distances to morphological variation, our conclusions were based on Procrustes distances and permutation tests, indicating that the degree of sexual shape dimorphism in the operculum was greater in DL than in LS.

## 4 Discussion

### 4.1 Sexual Dimorphism in Body Size

Compared with higher vertebrates, fishes exhibit more primitive, plastic, and diverse patterns of sexual differentiation. Fish morphology is highly sensitive to environmental influences, and sexual dimorphism is therefore a common phenotypic trait (Andersson 1994). In *T. strauchii* , the larger body size of males likely enhances mate pursuit ability, fertilization success, and competitive dominance, potentially conferring advantages in territory defense, mate acquisition, and offspring protection (Horne et al. 2020). The greater body length and caudal peduncle length of males may enhance their locomotor capacity, enabling them to occupy a dominant position during the breeding process (Sudasinghe 2025). Additionally, larger body size, larger head size, and greater caudal‐peduncle depth may contribute to improved locomotor and feeding performance in males.

Sexual dimorphism was observed in the body width of the DL population and the dorsal fin size of the LS population, with females exhibiting faster growth in these traits despite the overall larger body size of males. The abdomen provides space for gonad development and egg storage (Wootton and Smith 2014), while the dorsal fin contributes to swimming locomotion and the maintenance of balance (Webb 1984; Lauder and Drucker 2004). The rapid growth of these traits is a fundamental morphological characteristic that enables females to adapt for reproduction. After sexual maturation, strong sexual selection associated with mate pursuit likely drives males to allocate more energy to dorsal fin growth, thereby creating a growth inflection point that offsets the earlier growth advantage of females. In the Liutiao Stream population, males develop larger heads due to allometric growth in head height. During sexual maturation, they invest more resources in head robustness traits, causing head height to diverge significantly between the sexes later in development. For traits including caudal peduncle depth, eye diameter, and eye interval in Dacao Lake, as well as the remaining fin dimensions and eye diameter in Liutiao Stream, males are significantly larger but do not show allometric growth. We propose that these differences are established during the hatching or juvenile stage and are maintained through isometric growth, likely under genetic or hormonal control (Walker 1997).

### 4.2 Sexual Dimorphism in Eye Size

Variations in eye size are commonly observed among fish. Since larger eyes are positively correlated with improved visual acuity, they likely aid in foraging, predator avoidance, and mate identification (Andersson et al. 2024). Predation pressure also influences eye size within species, typically causing males to evolve smaller eyes while females retain larger eyes, resulting in female‐biased eye size dimorphism (Svanbäck and Johansson 2019). Conversely, when reproductive behaviors are mediated primarily by visual signals—such as in deep‐sea fishes, where enlarged male eyes substantially extend visual detection range—this pattern can be reversed, resulting in male‐biased eye size dimorphism (Vu et al. 2024). In the turbid waters of Liutiao Stream, suspended sediments and organic particles sharply reduce visibility. Given that reproductive success depends largely on males actively locating and identifying mates (Trivers 2017), a robust and sensitive visual system is likely advantageous in turbid habitats for detecting females and recognizing courtship signals at greater distances. As eye size is positively correlated with visual acuity in fishes (Caves et al. 2017), larger eyes may compensate for turbidity‐induced visual attenuation by increasing light flux (Tiarks et al. 2024). Thus, we hypothesize that, under the combined influence of sexual and natural selection, males may evolve relatively larger eyes than females. However, in the clear waters of Dacao Lake, high visibility likely allows males to detect and court mates over long distances even without large eyes. Additionally, the presence of predatory rainbow trout ( *Oncorhynchus mykiss* ) in this basin means that larger eyes may increase visibility and raise predation risk. Under strong predation pressure, the survival cost of having larger eyes likely exceeds any additional mating advantage they might provide. Therefore, we hypothesize that evolving smaller eyes may represent an optimal survival strategy for males in this habitat (Svanbäck and Johansson 2019). Furthermore, as a highly metabolically active organ, the eye requires substantial energy for its maintenance and development. Consequently, intra‐ and interspecific variation in eye size may be constrained by metabolic costs (Moran et al. 2015). Concurrently, structural constraints associated with head morphology, as well as allometric effects related to overall head size, may also influence relative eye size (Striedter and Northcutt 2006; Merlo et al. 2024).

### 4.3 Sexual Dimorphism in Operculum Shape

Sexual dimorphism in the operculum could improve reproductive success through sexual selection (Andersson 1994). The rounded operculum in males may help distribute external forces, thereby enhancing impact resistance and reducing injury during fights; this proposition should be tested in future behavioral studies. This shape may also potentially enlarge the opercular cavity, thereby supporting high‐oxygen‐demand activities such as courtship displays and prolonged female pursuit during breeding (Kareklas et al. 2022). The larger cavity may increase suction power, thereby aiding males in capturing larger and more active invertebrates (Scharnweber 2020). Moreover, males possess skin‐covered cartilaginous protrusions on the lateral ethmoid below the eye; the blunt operculum may enhance female perception of male attractiveness (Mori 1984) or intimidate rivals. Conversely, the narrow operculum of females may reduce ossification, thereby freeing energy for greater reproductive investment in egg development.

Furthermore, specific foraging environments may significantly influence the development and persistence of sexual dimorphism in fish (Jin et al. 2021; Table S13). The relatively stable oxbow‐lake habitat of Dacao Lake facilitates the establishment and defense of territories by males, which in turn increases male competition for mates and intensifies sexual selection (Endler 1980; Magurran and Seghers 1994). As a result, males likely evolve stronger opercula with greater muscle attachment to support combat behavior. In addition, the slow‐flowing, low‐elevation conditions provide higher dissolved oxygen levels, enabling both sexes to redirect conserved energy toward traits involved in sexual selection and competition. However, within the energetically demanding environment of Liutiao Stream, ecological pressures such as food availability and habitat utilization likely drive morphological evolution in this species (Choi et al. 2020). In fast‐flowing water, energy must be prioritized for resisting currents and maintaining basic metabolism. Under such conditions, males may adopt a “roving” strategy to find spawning‐ready females rather than a territorial “guarding” strategy (Fleming and Gross 1994; Gross 1996). Strong natural selection imposed by low dissolved oxygen also pushes both sexes toward shapes suited for efficient respiration (Chapman and Hulen 2001; Pörtner and Knust 2007). This leads to a more streamlined body and opercular morphology that maximize respiratory efficiency (Schaack and Chapman 2003), rather than traits useful for fighting. Therefore, operculum morphology becomes more similar between the sexes, resulting in less distinct sexual dimorphism in Liutiao Stream.

The discrepancy between the significant Procrustes distance and non‐significant Mahalanobis distance in our statistical analysis arises because Procrustes distance quantifies absolute shape differences under isotropic conditions, whereas Mahalanobis distance incorporates the within‐group covariance structure and assigns lower weight to shape variation along axes of high within‐group variability (Klingenberg and Monteiro 2005). This pattern suggests that habitat‐driven sexual dimorphism is expressed in morphometric traits (operculum) characterized by relatively high phenotypic plasticity or natural variability, rather than being highly canalized or evolutionarily conserved. The operculum appears sensitive to local environmental conditions, including water flow velocity and dissolved oxygen levels, which may in turn facilitate habitat‐specific morphological adaptation.

### 4.4 Geometric/Linear Morphometrics in Fish Studies

Investigations using geometric morphometrics and linear morphometrics across diverse fish species demonstrate that integrating these two approaches is critical for disentangling the complex interplay between morphology and adaptive evolution. For instance, linear measurements combined with 3D head truss analysis have revealed that head morphology in rock‐dwelling *Haplochromines* from Lake Victoria is significantly correlated with ecological variables. Specifically, head shape in rock‐dwelling taxa is closely linked to foraging mode, with species feeding on attached organisms exhibiting specialized cranial morphology associated with jaw musculature (Bouton et al. 2002). Geometric morphometric analyses have shown that body shape variation in Tanganyikan cichlids is strongly adaptive and closely associated with habitat preference. Species occupying deeper, calm waters exhibit a more streamlined body form suited to stable hydrodynamic environments, whereas those from shallow, wave‐washed habitats display body shapes better adapted to turbulent conditions (Clabaut et al. 2007). A combined application of linear and geometric morphometrics has demonstrated that, in strongly allometric, slender‐bodied species of the genus *Leporinus*, both methods can effectively distinguish closely related taxa after correcting for body size effects, with geometric morphometrics offering greater precision in detecting subtle morphological differences (Sidlauskas et al. 2011).

### 4.5 Prospects

In the future, landmark and semi‐landmark based methods could be employed to quantify the anterior head morphology of *T. strauchii* , facilitating the investigation of morphological associations between the operculum and the anterior head region. Additionally, geometric morphometrics can be applied to further explore interspecific morphological differences among sympatric fish species, including variations in the operculum, fins, caudal peduncle, and body proportions. Furthermore, classifying individuals into juvenile and adult age classes would help clarify the association between sexual dimorphism and allometric growth. Further work could integrate Computed Tomography (CT) and X‐ray imaging to examine differences between sexes in skull, vertebral, and finray structures. While the current study used 2D landmark methods, the potential application of 3D landmark approaches (Hallgrimsson et al. 2015) to study sexual dimorphism in *T. strauchii* remains to be explored.

### 4.6 Limitation

Captured individuals were anesthetized and then fixed and preserved in 10% formaldehyde for laboratory analysis, potentially causing tissue shrinkage and affecting morphometric measurements (Bayer and Counihan 2001; Martinez et al. 2013). However, relevant comparative studies on fishes have shown that 10% formalin fixation results in significantly less shrinkage than direct ethanol preservation (Paradis et al. 2007).

## 5 Conclusions

Sexual dimorphism in *T. strauchii* across two basins consistently shows male‐biased size dimorphism, particularly in the head (including the eyes and operculum) and caudal region. The most pronounced differences are observed in opercular shape and the narrowest part of the caudal peduncle. Predation pressure and water turbidity jointly drive sexual dimorphism in eye size, leading to larger eyes in males from Liutiao Stream but larger eyes in females from Dacao Lake. Additionally, sexual dimorphism in operculum shape is characterized by a narrow operculum in females and a rounded, blunt operculum in males, with the degree of dimorphism being more pronounced in Dacao Lake than in Liutiao Stream. Differences in the balance of survival strategies may be the main reason why operculum dimorphism varies between the two basins. In the stream habitat of Liutiao Stream, males may tend to use a “roving” rather than “guarding” reproductive strategy, and their specialized operculum shape likely supports the high respiratory efficiency required for this behavior. However, in the oxbow‐lake habitat of Dacao Lake, males rely on rounded, blunt opercula to cushion impacts during fights and to support high‐oxygen‐demand activities such as courtship, while females have narrow, elongated opercula that help direct more energy toward egg development. These results reveal the fine‐scale responses of fish sexual dimorphism to different selective forces. Furthermore, *T. strauchii* is under increasing threat, and habitat degradation, including flow velocity changes, water quality deterioration, and biological invasions, represents a key driver of population declines. Disturbances to natural habitats can disrupt adaptive morphological traits and sexual signaling systems, including eye‐size dimorphism and opercular morphology, thereby influencing male–male competition, mate choice, and reproductive success and ultimately increasing extinction risk. Strengthened conservation strategies for *T. strauchii* are therefore warranted, and the findings of this study provide a scientific basis for the conservation of this species and related freshwater fish.

## Author Contributions

**Yan Li:** data curation (lead), formal analysis (lead), methodology (lead), software (lead), visualization (lead), writing – original draft (lead), writing – review and editing (lead). **Ya‐Han Meng:** data curation (supporting), formal analysis (supporting), methodology (equal), software (supporting), validation (equal), writing – original draft (supporting), writing – review and editing (supporting). **Wei‐Zhen Gao:** conceptualization (equal), investigation (equal), methodology (equal), project administration (equal), resources (equal), validation (equal), visualization (equal), writing – original draft (supporting), writing – review and editing (equal). **Lei Shi:** conceptualization (lead), funding acquisition (lead), investigation (lead), methodology (lead), project administration (lead), resources (lead), supervision (lead), writing – original draft (equal), writing – review and editing (lead).

## Funding

This study was supported by the Third Xinjiang Scientific Expedition Program (grant no. 2022xjkk1200), the College Student Innovation and Entrepreneurship Training Program of Xinjiang Agricultural University (grant no. dxscx2025530).

## Ethics Statement

All experimental procedures involving animals were approved by the Animal Welfare and Ethics Committee of Xinjiang Agricultural University, Urumqi, Xinjiang, China (animal protocol number: 2023014).

## Consent

The authors have nothing to report.

## Conflicts of Interest

The authors declare no conflicts of interest.

## Supporting information


**Table S1:** Morphological measurements of *Triplophysa strauchii* from Dacao Lake (mm).
**Table S2:** Morphological measurements of *Triplophysa strauchii* from Liutiao Stream (mm).
**Figure S3:** Fish body wireframe diagram of female (A‐Dacao Lake, C‐Liutiao Stream) and male (B‐Dacao Lake, D‐Liutiao Stream).
**Figure S4:** PCA scatter plot (PC1 vs. PC2) of body morphology for the four groups of *Triplophysa strauchii* :female (A‐Dacao Lake, C‐Liutiao Stream) and male (B‐Dacao Lake, D‐Liutiao Stream).
**Figure S5:** PCA scatter plot (PC1 vs. PC2) of operculum morphology for the four groups of *Triplophysa strauchii* :female (A‐Dacao Lake, C‐Liutiao Stream) and male (B‐Dacao Lake, D‐Liutiao Stream).
**Table S6:** Procrustes ANOVA of operculum between sexes in *Triplophysa strauchii* from Dacao Lake—centroid size.
**Table S7:** Procrustes ANOVA of operculum between sexes in *Triplophysa strauchii* from Dacao Lake—shape.
**Table S8:** Procrustes ANOVA of operculum between sexes in *Triplophysa strauchii* from Liutiao Stream—centroid size.
**Table S9:** Procrustes ANOVA of operculum between sexes in *Triplophysa strauchii* from Liutiao Stream—shape.
**Table S10:** Procrustes ANOVA for assessment of digitization error—centroid size.
**Table S11:** Procrustes ANOVA for assessment of digitization error‐shape.
**Table S12:** Pairwise comparisons of sexual dimorphism in eye size between habitats.
**Table S13:** Habitat differences between Dacao Lake and Liutiao Stream.
**Table S14:** Schematic diagram of the statistical analysis workflow.

## Data Availability

All the required data are uploaded as Supporting Information.
