# Supplementary material for: Habitat‐Driven Sexual Dimorphism in Triplophysa strauchii Between Oxbow Lake and Stream: A Geometric Morphometric Analysis
Source: Ecol Evol. 2026 May 11;16(5):e73666. doi: 10.1002/ece3.73666 (PMC13160657; doi:10.1002/ece3.73666)
Supplement: Supplementary file 1 — Table S1: Morphological measurements of Triplophysa strauchii from Dacao Lake (mm). Table S2: Morphological measurements of Triplophysa strauchii from Liutiao Stream (mm). Figure S3: Fish body wireframe diagram of female (A‐Dacao Lake, C‐Liutiao Stream) and male (B‐Dacao Lake, D‐Liutiao Stream). Figure S4: PCA scatter plot (PC1 vs. PC2) of body morphology for the four groups of Triplophysa strauchii :female (A‐Dacao Lake, C‐Liutiao Stream) and male (B‐Dacao Lake, D‐Liutiao Stream). Figure S5: PCA scatter plot (PC1 vs. PC2) of operculum morphology for the four groups of Triplophysa strauchii :female (A‐Dacao Lake, C‐Liutiao Stream) and male (B‐Dacao Lake, D‐Liutiao Stream). Table S6: Procrustes ANOVA of operculum between sexes in Triplophysa strauchii from Dacao Lake—centroid size. Table S7: Procrustes ANOVA of operculum between sexes in Triplophysa strauchii from Dacao Lake—shape. Table S8: Procrustes ANOVA of operculum between sexes in Triplophysa strauchii from Liutiao Stream—centroid size. Table S9: Procrustes ANOVA of operculum between sexes in Triplophysa strauchii from Liutiao Stream—shape. Table S10: Procrustes ANOVA for assessment of digitization error—centroid size. Table S11: Procrustes ANOVA for assessment of digitization error‐shape. Table S12: Pairwise comparisons of sexual dimorphism in eye size between habitats. Table S13: Habitat differences between Dacao Lake and Liutiao Stream. Table S14: Schematic diagram of the statistical analysis workflow. [file ECE3-16-e73666-s001.docx]

Appendix

Table S1. Morphological measurements of *Triplophysa strauchii* from Dacao Lake **(mm)**

| Traits | Male  (n=87) | Female  (n=85) | Independent-Samples T Test | ANCOVA |
| --- | --- | --- | --- | --- |
| Body length, BL | 99.628±1.635 | 92.341±1.533 | ***t*=3.2514, *P* <0.05** | |
| Body weight, BW | 13.502±0.606 | 11.283±0.597 | ***t*=2.6085, *P* <0.05** | |
| Total length,TL | 117.178±1.826 | 109.178±1.765 | ***t*=3.1501, *P* <0.05** | |
| Head length, HL | 22.144±0.387 | 20.987±0.357 |  | *F*_1,172_=1.782,  *P* >0.05 |
| Long torso, LT | 46.925±1.065 | 43.244±0.736 |  | *F*_1,172_=0.193,  *P* >0.05 |
| Tail length, TAL | 32.072±0.622 | 30.025±0.582 |  | *F*_1,172_=0.699,  *P >*0.05 |
| Snout length, SNL | 9.941±0.430 | 8.672±0.189 |  | *F*_1,172_=1.017,  *P* >0.05 |
| **Eye diameter, ED** | **3.995±0.800** | **4.013±0.096** |  | ***F*_1,172_=11.7,**  ***P* <0.001** |
| Postorbital head length, PHL | 9.320±0.179 | 8.784±0.175 |  | *F*_1,172_=0.784, *P >*0.05 |
| Body depth, BD | 16.027±0.369 | 14.402±0.356 |  | *F*_1,172_=0.799*, P >*0.05 |
| Caudal-peduncle depth, CPD | 7.821±0.142 | 6.932±0.139 |  | ***F*_1,172_=8.95,**  ***P* <0.05** |
| Body width, BWI | 13.705±0.261 | 13.138±0.313 |  | ***F*_1,172_=4.979,**  ***P* <0.05** |
| Eye interval, EI | 9.330±0.173 | 9.030±0.187 |  | ***F*_1,172_=4.365,**  ***P* <0.05** |
| Dorsal fin length, DL | 18.422±0.429 | 17.158±0.345 |  | *F*_1,172_=0.37, *P >*0.05 |
| Caudal fin length, CL | 17.958±0.364 | 16.782±0.325 |  | *F*_1,172_=0.002, *P >*0.05 |
| Pelvic fin length , VL | 14.708±0.336 | 13.933±0.283 |  | *F*_1,172_=0.423, *P >*0.05 |
| Anal fin length, AL | 15.034±0.341 | 14.016±0.265 |  | *F*_1,172_=0.156, *P >*0.05 |
| Pectoral fin length, PL | 16.934±0.328 | 15.458±0.306 |  | *F*_1,172_=2.087, *P >*0.05 |
| Head height, HH | 13.394±0.280 | 11.998±0.248 |  | ***F*_1,172_=4.77,**  ***P* <0.05** |

Notes: Bold values indicate significant differences between groups (*P* <0.05 or *P* <0.001)

Table S2. Morphological measurements of *Triplophysa strauchii* from Liutiao Stream **(mm)**

| Traits | Male  (n=32) | Female  (n=60) | Independent-Samples T Test | ANCOVA |
| --- | --- | --- | --- | --- |
| Body length, BL | 91.642±8.679 | 82.697±8.729 | ***t*=-4.6994, *P* <0.001** | |
| Body weight, BW | 9.588±2.064 | 7.973±2.361 | ***t*=-3.3970, *P* <0.05** | |
| Total length,TL | 107.883±10.062 | 97.138±10.206 | ***t*=-4.8543, *P* <0.001** | |
| Head length, HL | 20.696±2.308 | 18.460±1.833 |  | *F*_1,92_=3.436 *P >*0.05 |
| Long torso, LT | 40.408±4.565 | 36.139±4.617 |  | *F*_1,92_=0.094, *P >*0.05 |
| Tail length, TAL | 29.671±2.979 | 27.098±3.492 |  | *F*_1,92_=0.536, *P >*0.05 |
| Snout length, SNL | 8.448±1.095 | 7.410±0.980 |  | *F*_1,92_=1.807, *P >*0.05 |
| **Eye diameter, ED** | **3.697±0.410** | **3.354±0.327** |  | ***F*_1,92_=5.191,**  ***P* <0.05** |
| Postorbital head length, PHL | 9.525±1.002 | 8.423±1.097 |  | *F*_1,92_=1.923, *P >*0.05 |
| Body depth, BD | 14.588±1.851 | 14.462±2.058 |  | *F*_1,92_=3.787*, P >*0.05 |
| Caudal-peduncle depth, CPD | 5.453±0.708 | 4.942±1.139 |  | *F*_1,92_=0.010, *P >*0.05 |
| Body width, BWI | 13.168±1.813 | 12.946±1.972 |  | *F*_1,92_=3.869, *P >*0.05 |
| Eye interval, EI | 8.137±0.795 | 7.498±0.699 |  | *F*_1,92_=2.144, *P >*0.05 |
| Dorsal fin length, DL | 18.110±1.512 | 15.927±1.895 |  | ***F*_1,92_=7.909,**  ***P* <0.05** |
| Caudal fin length, CL | 16.707±1.900 | 15.175±1.519 |  | *F*_1,92_=1.779, *P >*0.05 |
| Pelvic fin length , VL | 14.540±1.830 | 12.606±1.702 |  | ***F*_1,92_=4.928,**  ***P* <0.05** |
| Anal fin length, AL | 13.848±1.403 | 11.955±1.679 |  | ***F*_1,92_=6.584,**  ***P* <0.05** |
| Pectoral fin length, PL | 15.966±1.926 | 13.192±1.455 |  | ***F*_1,92_=31.589, *P* <0.001** |
| Head height, HH | 10.762±1.357 | 9.547±0.947 |  | ***F*_1,92_=8.688,**  ***P* <0.05** |

Notes: Bold values indicate significant differences between groups (*P* <0.05 or *P* <0.001)


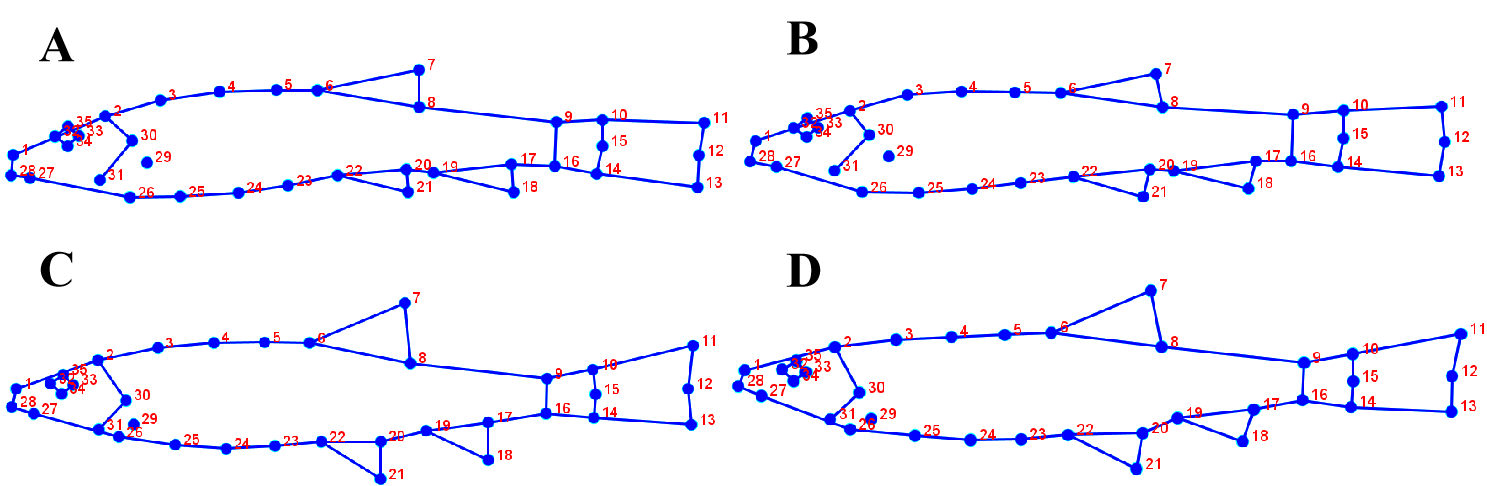


Figure S3. **Fish body wireframe diagram** of female ( A-Dacao Lake, C-Liutiao Stream)

and male ( B-Dacao Lake, D-Liutiao Stream)


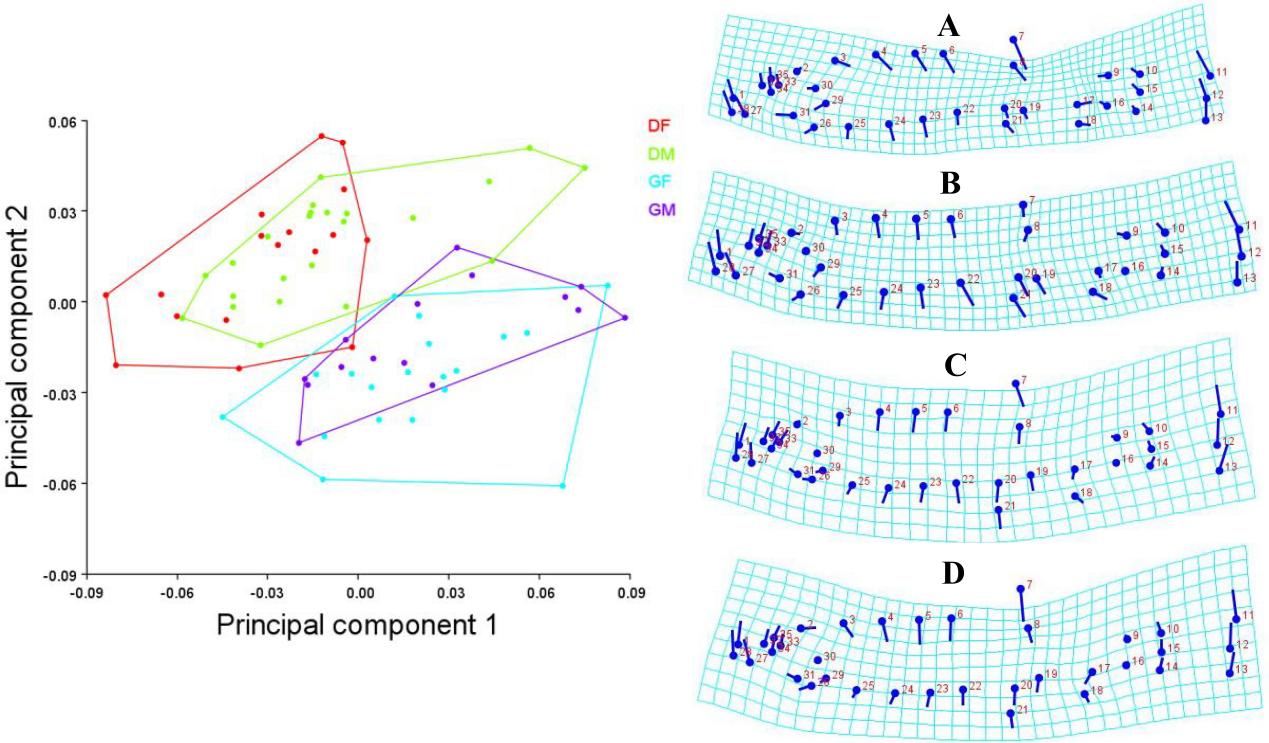


Figure S4. PCA scatter plot (PC1 vs. PC2) of body morphology for the four groups of Triplophysa *strauchii：*female ( A-Dacao Lake, C-Liutiao Stream) and male ( B-Dacao Lake, D-Liutiao Stream)

Notes: DF -Female *T. strauchii* from Dacao Lake DM-Male *T. strauchii* from Dacao Lake

GF-Female *T. strauchii* from Liutiao Stream GM-Male *T. strauchii* from Liutiao Stream


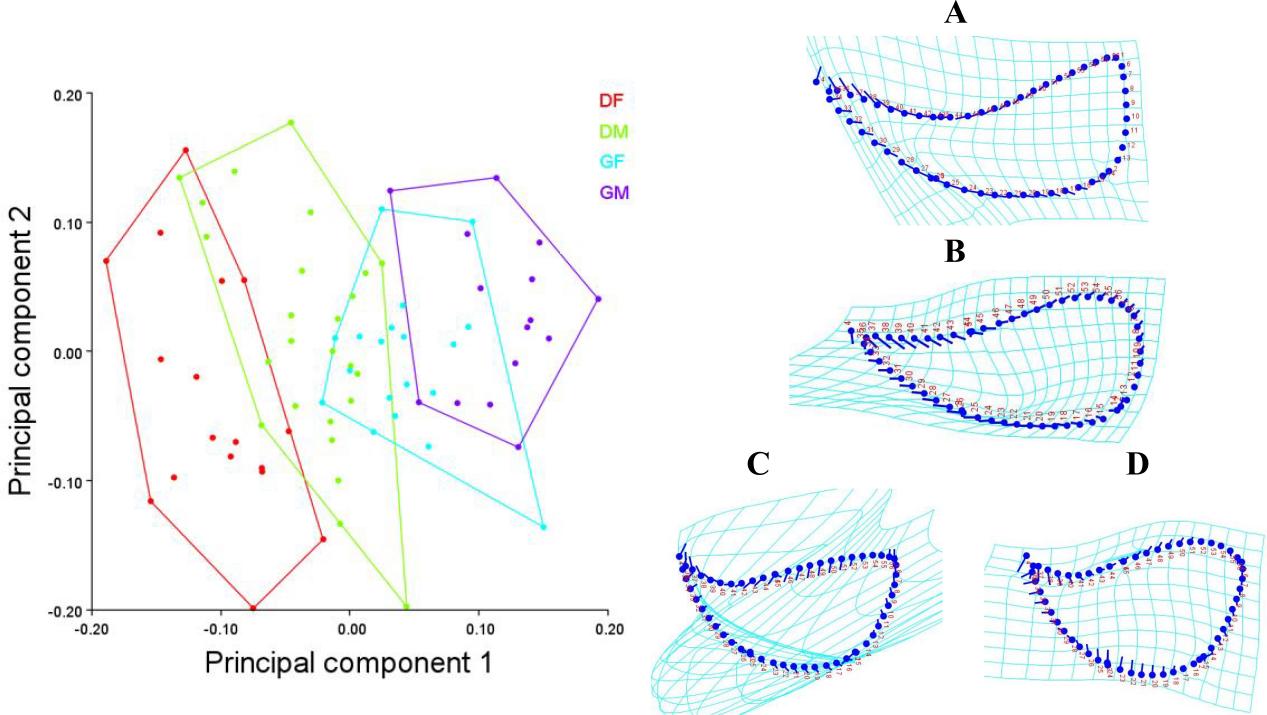


Figure S5. PCA scatter plot (PC1 vs. PC2) of operculum morphology for the four groups of Triplophysa *strauchii：*female ( A-Dacao Lake, C-Liutiao Stream) and male ( B-Dacao Lake, D-Liutiao Stream)

Notes: DF -Female *T. strauchii* from Dacao Lake DM-Male *T. strauchii* from Dacao Lake

GF-Female *T. strauchii* from Liutiao Stream GM-Male *T. strauchii* from Liutiao Stream

Table S6. Procrustes ANOVA of operculum between sexes in *Triplophysa strauchii* from Dacao Lake - centroid size

| Effect | SS | MS | df | F | *P* (param.) |
| --- | --- | --- | --- | --- | --- |
| Individual | 1.765337 | 0.110334 | 16 | 0.46 | *P* >0.05 |
| Sex | 0.498761 | 0.498761 | 1 | 2.09 | *P* >0.05 |
| Ind * Sex | 3.813964 | 0.238373 | 16 |  |  |

Table S7. Procrustes ANOVA of operculum between sexes in *Triplophysa strauchii* from Dacao Lake - shape

| Effect | SS | MS | df | F | *P* (param.) |
| --- | --- | --- | --- | --- | --- |
| Individual | 0.31347468 | 0.0001814090 | 1728 | 1.41 | *P* <.0001 |
| Sex | 0.11985460 | 0.0011097648 | 108 | 8.64 | *P* <.0001 |
| Ind * Sex | 0.22204664 | 0.0001284992 | 1728 |  |  |

Table S8. Procrustes ANOVA of operculum between sexes in *Triplophysa strauchii* from

Liutiao Stream - centroid size

| Effect | SS | MS | df | F | *P* (param.) |
| --- | --- | --- | --- | --- | --- |
| Individual | 1.917379 | 0.136956 | 14 | 0.99 | *P* >0.05 |
| Sex | 0.071024 | 0.071024 | 1 | 0.51 | *P* >0.05 |
| Ind * Sex | 1.939710 | 0.138551 | 14 |  |  |

Table S9. Procrustes ANOVA of operculum between sexes in *Triplophysa strauchii* from

Liutiao Stream - shape

| Effect | SS | MS | df | F | *P* (param.) |
| --- | --- | --- | --- | --- | --- |
| Individual | 0.20876149 | 0.0001380698 | 1512 | 0.98 | *P* >0.05 |
| Sex | 0.06605751 | 0.0006116437 | 108 | 4.35 | *P* <.0001 |
| Ind * Sex | 0.21239520 | 0.0001404730 | 1512 |  |  |

Table S10. Procrustes ANOVA for assessment of digitization error - centroid size

| Effect | SS | MS | df | F | *P* (param.) |
| --- | --- | --- | --- | --- | --- |
| Individual | 164.209209 | 8.642590 | 19 | 33.96 | <.0001 |
| Error 1 | 5.090331 | 0.254517 | 20 |  |  |

Table S11. Procrustes ANOVA for assessment of digitization error - shape

| Effect | SS | MS | df | F | *P* (param.) |
| --- | --- | --- | --- | --- | --- |
| Individual | 0.10820891 | 0.0000862910 | 1254 | 16.03 | <.0001 |
| Error 1 | 0.00710460 | 0.0000053823 | 1320 |  |  |

Table S12. Pairwise comparisons of sexual dimorphism in eye size between habitats

| Habitat | Indicator type | Distance | *P*-value |
| --- | --- | --- | --- |
| Dacao Lake (DL) | Size-related (predicted value) | 0.252682 | <0.05 |
|  | Size-corrected (residual) | 0.2718693 | <0.05 |
| Liutiao Stream (LS) | Size-related (predicted value) | 0.2035445 | **<0.001** |
|  | Size-corrected (residual) | 0.1389971 | **=0.05** |

Table S13. Habitat differences between Dacao Lake and Liutiao Stream

|  | **Dacao Lake (DL)** | **Liutiao Stream (LS)** |
| --- | --- | --- |
| habitat | oxbow lake | **stream** |
| flow velocity | **slow-flowing** | **fast-flowing** |
| turbidity | **clear waters** | **turbid waters** |
| **dissolved oxygen** | **high** | **low** |
| substrate | minerotrophic peatland dominated by aquatic plants | sediment-bottomed stream |

Table S14. Schematic diagram of the statistical analysis workflow

**Statistical Analysis**

**Traditional Morphometric Analysis**

**Geometric Morphometric Analysis**

**Independent-Samples T Tests +ANCOVA**

**RMA-Allometric Growth**

**GPA (Standardization) +PCA**

**PMANOVA-*P*-value**

**CVA-Quantify SD**
